# Tag-Trigger-Consolidation: A Model of Early and Late Long-Term-Potentiation and Depression

**DOI:** 10.1371/journal.pcbi.1000248

**Published:** 2008-12-26

**Authors:** Claudia Clopath, Lorric Ziegler, Eleni Vasilaki, Lars Büsing, Wulfram Gerstner

**Affiliations:** Laboratory of Computational Neuroscience, Brain-Mind Institute and School of Computer and Communication Sciences, Ecole Polytechnique Fédérale de Lausanne, Lausanne, Switzerland; UFR Biomédicale de l'Université René Descartes, France

## Abstract

Changes in synaptic efficacies need to be long-lasting in order to serve as a substrate for memory. Experimentally, synaptic plasticity exhibits phases covering the induction of long-term potentiation and depression (LTP/LTD) during the early phase of synaptic plasticity, the setting of synaptic tags, a trigger process for protein synthesis, and a slow transition leading to synaptic consolidation during the late phase of synaptic plasticity. We present a mathematical model that describes these different phases of synaptic plasticity. The model explains a large body of experimental data on synaptic tagging and capture, cross-tagging, and the late phases of LTP and LTD. Moreover, the model accounts for the dependence of LTP and LTD induction on voltage and presynaptic stimulation frequency. The stabilization of potentiated synapses during the transition from early to late LTP occurs by protein synthesis dynamics that are shared by groups of synapses. The functional consequence of this shared process is that previously stabilized patterns of strong or weak synapses onto the same postsynaptic neuron are well protected against later changes induced by LTP/LTD protocols at individual synapses.

## Author Summary

Humans and animals learn by changing the strength of connections between neurons, a phenomenon called synaptic plasticity. These changes can be induced by rather short stimuli (lasting sometimes only a few seconds) but should then be stable for months or years in order to be useful for long-term memory. Experimentalists have shown that synapses undergo a sequence of steps that transforms the rapid change during the early phase of synaptic plasticity into a stable memory trace in the late phase. In this paper we introduce a model with a small number of equations that can describe the phenomena of induction of synaptic changes during the early phase of synaptic plasticity, the trigger process for protein synthesis, and the final stabilization. The model covers a broad range of experimental phenomena known as tagging experiments and makes testable predictions. The ability to model the stabilization of synapses is crucial to understand learning and memory processes in animals and humans and a necessary ingredient for any large-scale model of the brain.

## Introduction

Changes in the connection strength between neurons in response to appropriate stimulation are thought to be the physiological basis for learning and memory formation [1],[2]. A minimal requirement for proper memory function is that these changes, once they are induced, persist for a long time. For several decades, experimentalists have therefore focused on Long-Term Potentiation (LTP) and Long-Term Depression (LTD) of synapses in hippocampus [3],[4] and cortical areas [5],[6]. LTP can be induced at groups of synapses by strong ‘tetanic’ high-frequency stimulation of the presynaptic pathway [3] while stimulation at lower frequency leads to LTD Dudek92. Both LTP and LTD can also be induced at a single synapse or a small number of synaptic contacts if presynaptic activity is paired with either a depolarization of the postsynaptic membrane [5],[7] or tightly timed postsynaptic spikes [8],[9].

While the induction protocol for LTP and LTD is often as short as a few seconds, the changes in synaptic efficacy persist for much longer [9]. In typical slice experiments on LTP [and similarly for LTD or Spike-Timing Dependent Plasticity (STDP)] the persistence of the change is monitored for 30 minutes to 1 hour. Accumulating evidence suggests, however, that after this early phase of LTP (E-LTP) different biochemical processes set in that are necessary for the further maintenance of potentiated synapses during the late phase of LTP (L-LTP) [10],[11]. For an understanding of the transition from early to late LTP, the concept of ‘synaptic tagging and capture’ has become influential [12],[13]. During induction of the early phase of LTP, each potentiated synapse sets a tag that marks that it has received a specific afferent signal. A candidate molecule, involved in the tag signaling LTP induction in apical dendrites of hippocampal neurons, is the calcium-calmodulin dependent kinase II (CaMKII) [13]. Newly synthesized plasticity-related proteins are ‘captured’ by the tagged synapse and transform E-LTP into L-LTP that can be maintained over hours or days. A candidate protein involved in the maintenance of potentiated hippocampal synapses is the protein kinase M*ζ* (PKM*ζ*) [11],[14].

The stabilization and maintenance of potentiated synapses poses a number of theoretical challenges. First, on the level of single synapses we must require synaptic strength to remain stable, despite the fact that AMPA channels in the postsynaptic membrane are continuously exchanged and recycled [15]–[17]. Thus the synapse is not ‘frozen’ but part of a dynamic loop. Second, on the level of neuronal representation in cortical areas, one finds representations of input features that are stable but at the same time sufficiently plastic to adjust to new situations [18]. In the theoretical community, this paradox has been termed the stability-plasticity dilemma in unsupervised learning [19]. Third, humans keep the ability to memorize events during adulthood, but can also remember earlier episodes years back. However, continued learning of new patterns in theoretical models of associative memory networks forces the erasure or ‘overwriting’ of old ones, the so-called palimpsest property [20],[21]. In the context of continued learning, theoretical arguments show that synaptic plasticity on multiple time scales cannot prevent, but at most delay the erasure of memories in the presence of ongoing synaptic activity [22]. This suggests that additional mechanisms are necessary to further protect existing memories and ‘gate’ the learning of new ones.

Despite these challenges for the long-term stability of synapses, most classical models of synaptic plasticity focus on the induction and early phase of LTP or LTD and completely ignore the question of maintenance. Traditional models of associative memories separate the learning phase from the retrieval phase [23] and the same holds for standard models of STDP [24]–[26]. Detailed biophysical models of LTP and LTD describe calcium dynamics and Calcium/Calmodulin-Dependent Protein Kinase II (CaMKII) phosphorylation during the induction and early phase of LTP [27]–[29]. While these models show that switches built of CaMKII proteins can be stable for years, they do not address aspects of tagging leading to heterosynaptic interaction during L-LTP and L-LTD. Moreover, while CaMKII phosphorylation is necessary for induction of LTP and mediate tags in the apical dendrites of hippocampal CA1 neurons [30], it is less clear whether it is necessary for its maintenance [31]. On the other hand protein kinase M*ζ* is essential for maintenance of some synapse types [11],[13],[14] but the same molecule is potentially relevant for *induction* in others [30].

We wondered whether a simple model that connects the process of LTP induction with that of maintenance would account for experimental results on tagging and ‘cross-tagging’ [11]–[13],[32] without specific assumptions about the (partially unknown) molecular pathways involved in the maintenance process. If so, the model should allow us to discuss functional consequences that are generic to the tagging hypothesis independent of the details of a biophysical implementation in the cell. Even though we believe that the model principles are more general, we focus on synapses from the Schaffer-Collaterals onto the CA1 neurons in hippocampus as an experimentally well-studied reference system for synaptic plasticity. Since typical tagging experiments involve the extracellular stimulation of one or several *groups* of synapses (rather than single synapses), our model of early and late LTP/LTD is developed in the context of a neuron model with hundreds of synapses. The application of the principles of synaptic consolidation to experiments inducing E-LTP/E-LTD at *single* synapses is considered in the discussion section.

## Results

We study a model with a large number of synapses *i* onto a single postsynaptic neuron. To be specific, we think of a pyramidal neuron in the CA1 area of hippocampus. Our model combines features of traditional models for the *induction* of potentiation [24]–[26], [33]–[36] with a simple description of tagging and synthesis of plasticity related proteins that finally lead to the *maintenance* of the induced changes. The section is organized as follows: We first introduce the essential components of the model step by step (‘Constructing the Model’). We then test the performance of the model with a set of stimuli typically used to induce long-term changes of synapses (‘Testing the Model’).

### Constructing the Model

**Figure 1:**
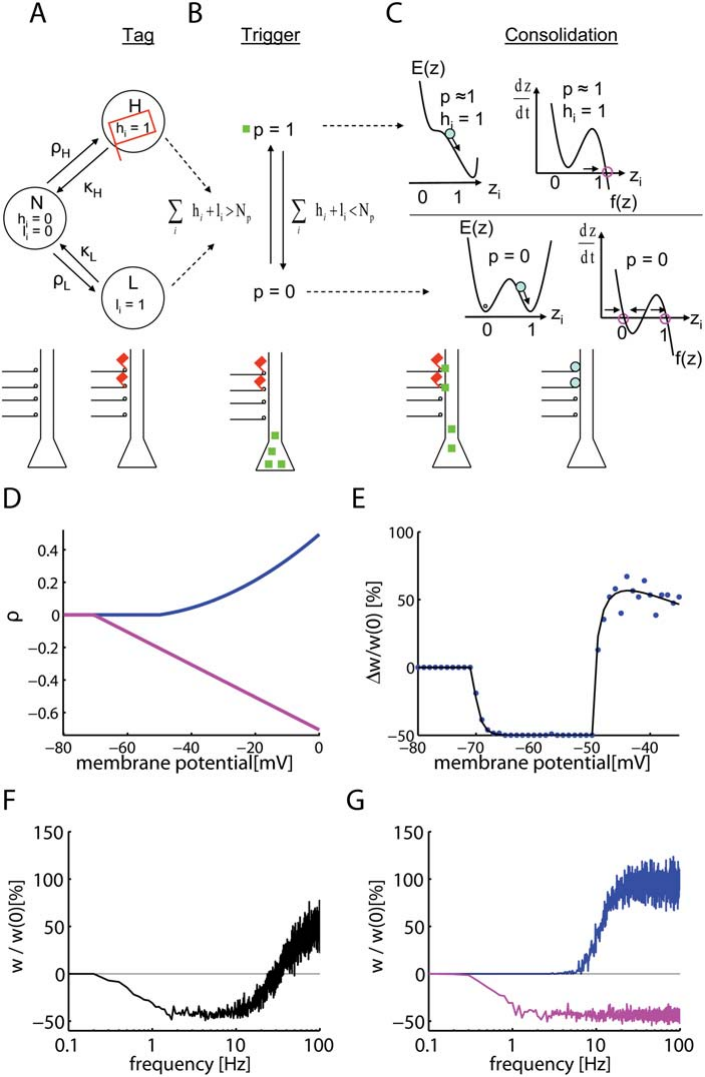
The three components of the Tag-Trigger-Consolidation (TagTriC) model. (A) A synapse can be in the non-tagged state N, the high state H or the low state L. A synapse *i* in H (or L) has a tag *h_i_* = 1 (or *l_i_* = 1, respectively). Transitions to a tagged state occur with rates *ρ_H_* for potentiation and *ρ_L_* for depression. The tag *h_i_* = 1 is indicated by a red flag in both the flow graph and the schematic drawing below. (B) Synthesis of plasticity related proteins *p* (green squares) is triggered if the total number of set tags is larger than a critical number *N_p_*. If the trigger threshold *N_p_* is not reached, the protein concentration decays back to zero. (C) The consolidation dynamics can be visualized as downward motion in a potential surface *E*(*z*). The function *f*(*z*) (shown to the right) is the derivative of *E* and characterizes the dynamics *dz*/*dt* = *f*(*z*). If a tag is set at the synapse (*h_i_* = 1) and protein synthesis has been triggered (*p*≈1), the dynamics can be imagined as downward motion into the right well of the potential *E*(*z*). In this case, *z*≈1 is the only fixed point of the dynamics (magenta circle). In the absence of tags (*h_i_* = *l_i_* = 0, below) the consolidation variable *z_i_* of synapse *i* is bistable and approaches (direction of flow indicated by arrows) stable fixed points at *z_i_* = 0 or *z_i_* = 1 (magenta circles). The steps of synaptic tagging and capture are indicated immediately below the flow diagram. (D) The tagging rates for depression (−*ρ_L_*,(magenta)) and for potentiation *ρ_H_* (blue) are shown as a function of the clamped voltage under the assumption that a presynaptic spike has arrived less than 1 millisecond before. Note that for depression we plot the negative rate −*ρ_L_* rather than *ρ_L_* to emphasize the fact that depression leads to a down-scaling of the synapse. (E) Voltage dependence of early LTP and LTD. The weight change Δ*w*/*w*(0) induced by a stimulation of 100 synapses at 2 Hz during 50 s while the postsynaptic voltage is clamped is shown as a function of voltage. The percent change Δ*w*/*w̅* in simulations (circles) of LTP/LTD induction experiments can be predicted from a theory (solid line) based on the difference in transition rates *ρ_H_*−*ρ_L_*. The simulation reflects the voltage dependence seen in experiments [5],[39]. (F,G) Frequency dependence of early LTP and LTD. Simultaneous stimulation of 100 synapses by 3 trains (separated by 5 min) of 100 pulses at rates ranging 0.03 to 100 Hz shows LTD at low frequencies and LTP at frequencies above 30 Hz. (G) If LTP is blocked in the model, LTD (pink line) occurs up to high frequencies as in experiments [7]. Blue line: LTP with blocked of LTD.

Our model contains three elements, Figure 1. The first one sets the *tag* during the induction of E-LTP or E-LTD. A tag is indicated by a value *h* = 1 for LTP or *l* = 1 for LTD. In the absence of tags we have *h* = *l* = 0. The second one describes the process that *triggers* the synthesis of plasticity related proteins. The final component describes the up-regulation of a maintenance-related process from a low value (*z* = 0) to a high value (*z*≈1). The dynamics of this component is intrinsically bistable and leads to a *consolidation* of the previously induced change at the labeled synapses upon interaction with the protein *p* (‘protein capture’). The total change Δ*w* of the synaptic strength reported in experiments contains contributions [13] of the early components *l* and *h* as well as the late component *z*. Since the model describes a sequence of three steps ‘Tag-Trigger-Consolidation’ we call it in the following the TagTriC-Model (Figure 1).

### Tag and Induction of LTP/LTD

Results from minimal stimulation protocols which putatively activate only a single synapse suggest that the induction of LTP is a switch-like process [7],[37]. We therefore model individual synapses as discrete quantities that can switch, during the induction of LTP, from an initial ‘non-tagged state’ (N) to a ‘high state’ (H) with a transition rate *ρ_H_* that depends on the induction protocol. Similarly, induction of LTD moves the synapse from the initial non-tagged state (N) to a ‘low state’ (L) at a rate *ρ_L_*. If synapse *i* is in the high state, the synaptic variable *h_i_* is equal to one. If it is in the low state, another local variable *l_i_* is set to one. These local variables *h_i_* and *l_i_* do not only control the weight of the synapse during E-LTP and E-LTD, but also serve as ‘tags’ for up- or down-regulation of the synapse. Tags reset to zero stochastically with a rate *k_h_* and *k_l_*, respectively. If both tags are zero, the synapse is in the non-tagged state N. Since the synapse is either up-regulated OR down-regulated, at most one of the tags can be non-zero (Figure 1A).

The stochastic transitions from the initial state N with *h_i_* = 0 and *l_i_* = 0 to the down-regulated state *l_i_* = 1 or an upregulated state *h_i_* = 1 depend in a Hebbian manner on presynaptic activity and the state of the postsynaptic neuron. In the absence of presynaptic activity, the LTD rate *ρ_L_* vanishes. Presynaptic activity combined with a time-averaged membrane potential *u̅* above a critical value *ϑ*_LTD_ leads in the TagTriC model to a LTD transition rate *ρ_L_* proportional to [*u̅*(*t*)−*ϑ*_LTD_]. For a transition from the initial state to the high state, we require in addition that the *momentary* membrane potential is above a second threshold *ϑ*_LTP_. Hence the transition rate *ρ_H_* is proportional to [*u̅*(*t*)−*ϑ*_LTD_][*u*−*ϑ*_LTP_] whenever these threshold conditions are satisfied; see Methods for details.

Our assumptions regarding the transition rates essentially summarize the qualitative voltage dependence seen in the Artola-Bröcher-Singer experiments [5]. Indeed, when 100 synapses in the TagTriC model are stimulated at low frequency during 50 seconds while the membrane voltage is kept fixed at different values (Figure 1D), the total weight change summed across all synapses exhibits LTD at low voltage and LTP at high voltage [38],[39]. As expected, the resulting weight changes in the simulations of Figure 1E reflect the voltage dependence of the transition rates in Figure 1D.

### Trigger for Protein Synthesis

Previously induced LTP or LTD needs to be consolidated in order to last for more than one hour. Consolidation requires that protein synthesis is triggered. Experimental evidence indicates that triggering of protein synthesis needs the presence of neuromodulators such as dopamine (in the apical CA1 region) or other modulators (in other regions). In typical tagging experiments, extracellular stimulation co-stimulates dopaminergic input leading to a phasic dopamine signal [13],[40]. In our model, induction of E-LTP or E-LTD through appropriate stimulation protocols changes the synaptic efficacy and sets tags at the modified synapses, both described by the variables *h_i_* = 1 or *l_i_* = 1. Protein synthesis in the model is triggered (see methods for details) if the total number of tags Σ*_i_*(*h_i_*+*l_i_*) (which indirectly reflects the phasic dopamine signal) reaches a threshold *N_p_* which depends on the level of background dopamine (and other neuromodulators). More specifically, *N_p_* decreases with the concentration of background dopamine so that the presence of dopamine facilitates the trigger process [32].

If the trigger criterion is satisfied, the concentration *p* of synthesized plasticity related proteins approaches with rate *k_p_* a value close to one. If the number of tags falls below the threshold *N_p_*, the protein concentration *p* decays with a time constant *τ_p_* back to zero. Further details on the role of the trigger threshold and its relation to neuromodulators can be found in the discussion section.

### Consolidation and Late LTP

The total weight *w_i_* of a synapse *i* depends on the present value of the tags *h_i_* or *l_i_* as well as on its long-term value *z_i_*. The slow variable *z_i_* is a continuous variable with one or two stable states described by a generic model of bistable switches, that could be implemented by suitable auto-catalytic processes [16]. While the concentration *p* of plasticity related proteins is zero, the variable *z_i_* has two stable states at *z_i_* = 0 and *z_i_* = 1, respectively. If the protein concentration takes a value of *p*≈1, one of the stable states disappears and, depending on the tag that was set, the long term-value of the synapse can be up- or down-regulated; see methods and Figure 1C for details.

In order to illustrate the mechanism of induction of L-LTP, let us suppose that the synapse has been initially close to the state *z_i_* = 0. The dynamics of the synapse can be imagined as downward motion in a ‘potential’ *E*. The current stable state of the synapse is at the bottom of the left well in the potential pictured in Figure 1C. We assume that during a subsequent LTP induction protocol the synapse has been tagged with *h_i_* = 1 and that the total number of tags set during the LTP induction protocol surpasses the trigger threshold *N_p_*. If the protein concentration *p* approaches one, the potential surface is tilted so that the synapse now moves towards the remaining minimum at *z*≈1. After decay of the tags, *p* returns to zero, and we are back to the original potential, but now with the synapse trapped in the state *z* = 1. It can be maintained in this state for a long time, until another strong tagging event occurs during which the synapse is tagged with *l_i_* = 1 as a result of LTD induction. In this case the potential surface can be tilted towards the left so that the only equilibrium point is at *z* = 0. Since consolidation is typically studied in animals that are more than 20 days old [13], we assume that before the beginning of the experiment 30 percent of the synapses are already in the upregulated state *z* = 1 and the remaining 70 percent in the state *z* = 0; see also [7]. Because of the bistable dynamics of consolidation, only synapses that are initially in the upregulated state *z* = 1 can undergo L-LTD and only synapses that start from *z* = 0 can undergo L-LTP; compare [7]. Note, however, that tags for potentiation and depression can be set independently of the value of *z*. We may speculate that the variable *z* is related to the activity of PKM*ζ* [11],[14], or to the self-sustained clustering of AMPA receptors [41], but the exact biochemical signaling chain is irrelevant for the functional consequences of the model discussed in the results section. In our model, the bistable dynamics of the *z*-variable captures the essence of synaptic persistence despite molecular turnover [15],[16],[28] and mobility of AMPA receptors [41].

### Tests of the Model

The TagTriC model has been tested on a series of stimulation protocols that reflect induction of LTP and LTD as well as the consolidation of plasticity events.

### Induction of Synaptic Changes

A typical LTP induction experiment starts with extracellular stimulation of a bundle of presynaptic fibers (i.e., the Schaffer collaterals leading from CA3 to CA1) that activate a large number (typically hundreds [13]) of presynaptic terminals. With an extracellular probe electrode placed close to one of the postsynaptic neurons, a change in synaptic efficacy is measured via the amplitude (or initial slope) of the evoked postsynaptic potential, representing the total response summed across all the stimulated synapses. In our simulations, we mimic these experiments by simultaneous stimulation of 100 synapses. The state of the postsynaptic neuron is described by the adaptive exponential integrate-and-fire model [42] and can be manipulated by current injection.

In a preliminary set of simulation experiments done with presynaptic stimulation alone (no manipulation of the postsynaptic neuron), the TagTriC model exhibits LTD or LTP depending on the frequency of the presynaptic stimulation (Figure 1F) in agreement with experimental results [4],[43]. Moreover, under the assumption that LTP has been blocked pharmacologically (*ρ_H_* = 0 in the model), our model shows LTD even for high stimulation frequencies (Figure 1G). This stems from the fact that LTD and LTP are represented in the TagTriC model by two independent pathways (Figure 1A) which are under control condition in competition with each other, but show up individually if one of the paths is blocked [43]. Together with the voltage dependence of Figure 1E, the above simulation results indicate that our model of LTP and LTD induction can account for a range of experiments on excitatory synapses in the hippocampal CA1 region, in particular, voltage and frequency dependence.

### Consolidation of Synaptic Changes

**Figure 2:**
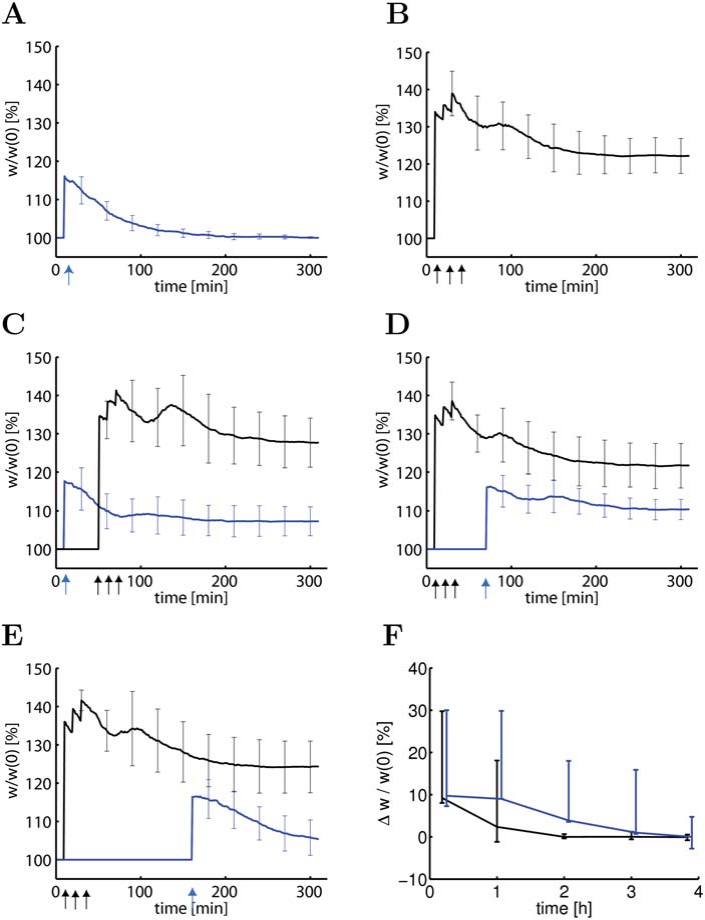
The model accounts for tagging paradigms. (A) A weak tetanus (21 pulses at 100 Hz) applied at a group of 100 synapses at *t* = 10 min (arrow) leads to an increased connection weight (*w*/*w*(0), blue line) that decays back to baseline. (B) A strong tetanus (100 pulses at 100 Hz repeated three times, arrows) leads to late LTP that is sustained for 5 hours (black line). (C) If the weak tetanus (blue arrow) in a first group of synapses is followed thirty minutes later by a strong tetanus (black arrows) in a second group of synapses, the weights in the first group (blue line) and the second group (black line) are stabilized above baseline. (D) Stimulating a group of synapses by a weak tetanus (blue arrow) 30 minutes *after* the end of the strong tetanic stimulation of a second group also leads to stabilization of the weights in both groups above baseline. (E) If the weak tetanic stimulation occurs 2 hours after the strong tetanic stimulation of the other group, only synapses in the strongly stimulated group will be stabilized (black line), but not those in the weakly stimulated group (blue line). (F) Fraction of stabilized weights Δ*w*/*w*(0) in the weakly stimulated group measured 10 hours after induction of LTP as a function of the time difference between the weak stimulation and the end of the strong tetanic stimulation in the second group. Blue line: normal set of parameters (*N_p_* = 40). Black line: protein trigger threshold increased to *N_p_* = 60. In panels A–E, lines indicate the result averaged over 10 repetitions of the simulation experiments and bars standard deviation. In panel F, line indicates the result averaged over 100 repetitions. 90 of the 100 individual trials stayed within the bounds indicated by the error bars.

In order to study whether consolidation of synaptic changes in our model follows the time course seen in experiments, we simulate standard experimental stimulation protocols [12],[13]. A weak tetanus consisting of a stimulation of 100 synapses at 100 Hz for 0.2 seconds (21 pulses) leads in our model to the induction of LTP (change by +15 percent) which decays back to baseline over the time course of two hours (Figure 2A). Thus, after the early phase of LTP the synapses are not consolidated. A stronger stimulus consisting of stimulating the same group of hundred synapses by 100 pulses at 100 Hz (repeated 3 times every 10 minutes) yields stronger LTP that consolidates and remains elevated (weight change by 22±5 percent) for as long as the simulations are continued (more than 10 hours, only the first 5 hours are shown in Figure 2B). Thus our model exhibits a transition from early to late LTP if E-LTP is induced by the strong tetanic stimulation protocol, but not the weak one, consistent with results in experiments [12],[13]. If, however, the weak tetanus at a first group of 100 synapses is given 30 minutes before or after a strong tetanus at a second group of 100 synapses, the synapses in both the weakly and strongly stimulated groups are consolidated (Figure 2C and 2D). If the weak tetanus in group one is given 120 minutes after the strong tetanus in group two, then consolidation of the synapses in the weakly stimulated group does not occur (Figure 2E). Thus our model exhibits a time course of heterosynaptic interaction between the two groups of synapses as reported in classical tagging experiments [12],[13].

An advantage of a modeling approach is that we can study the dependence of the heterosynaptic interaction between the two groups of synapses upon model parameters. A critical parameter in the model is the trigger threshold *N_p_* that needs to be reached in order to start protein synthesis (Figure 1B). With our standard choice of parameters, where *N_p_* = 40, we can plot the consolidated weight change Δ*w*/*w*(0) in the weakly stimulated group (measured 10 hours after the induction) as a function of the time difference between the stimulation of the group receiving the strong tetanus and that receiving the weak tetanus. The curve in Figure 2F shows that for a time difference up to 1 hour there is significant interaction between the two groups of synapses leading to synaptic consolidation, whereas for time differences beyond 2 hours this is no longer the case. If the trigger threshold is increased to *N_p_* = 60 (corresponding to less available neuromodulator), then the maximal time difference that still yields L-LTP in the weakly stimulated group of synapses is reduced to about 20 minutes (Figure 2F) whereas a reduction of *N_p_* yields an increased time window of interaction (data not shown). If *N_p_* is reduced much further, the weak tetanus alone will be sufficient to allow a transition from the early to the late phase of LTP. We speculate that *N_p_* could depend on the age of the animal as well as on the background level of dopamine or other neuromodulators so as to enable a tuning of the degree of plasticity (see discussion for details).

### LTD and Cross-Tagging

**Figure 3:**
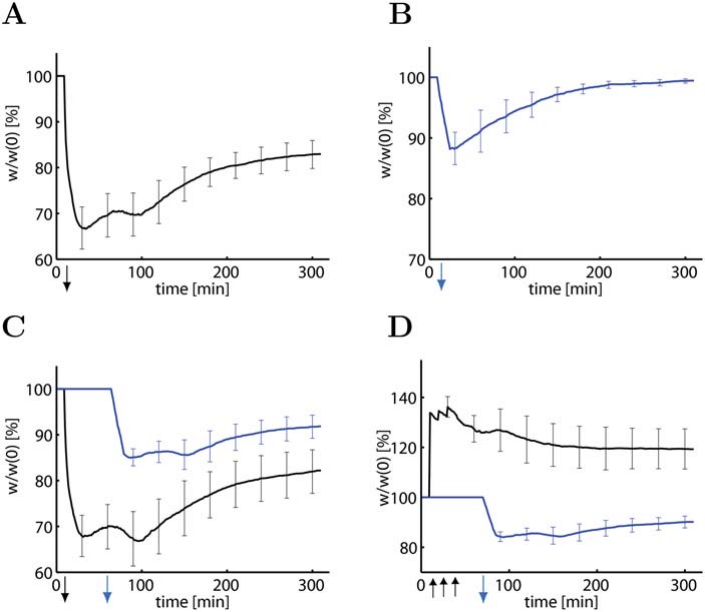
The model accounts for cross-tagging between LTP and LTD. (A) A strong low-frequency stimulus (3 pulses at 20 Hz, repeated 900 times every second) applied to a group of *N* = 100 synapses induces LTD with mean weights (*w*/*w*(0)) stabilized at 83±3% of initial value after 5 hours (black line). (B) A weak low-frequency stimulus (1 pulse repeated 900 times at 1 Hz) induces early LTD, which is not consolidated. (C) If the weak low-frequency stimulus is applied 30 minutes after a second group of synapses has received the strong low-frequency protocol, the weights in both groups (blue, weak stimulus; black, strong stimulus) are consolidated at values below baseline. (D) Consolidation of LTD in the group receiving weak low-frequency stimulation (blue line) also happens if induction occurs 30 minutes after stimulating a second group of synapses with a strong tetanic protocol (see Figure 2) inducing LTP (black line). Downward arrows indicated the period of weak (blue arrow) or strong (black arrow) low-frequency protocols. The black upward arrows indicate strong tetanic stimulation. Lines show mean results, averaged over 10 repetitions of the simulation experiment. Error bars are standard deviation.

We consider two experimental protocols known to induce LTD—a weak low-frequency protocol consisting of 900 pulses at 1 Hz and a strong low-frequency protocol consisting of 900 repetitions at 1 Hz of a short burst of three pulses at 20 Hz. This strong low-frequency protocol applied to 100 model synapses leads to a significant level of LTD (reduction of weights to 70±4 percent of initial value) which is consolidated 5 hours later at a level of 83±3 percent of initial value. If a group of 100 synapses is stimulated with the weak low-frequency protocol, an early phase of LTD is induced that is not consolidated but decays over the time course of 3 hours (Figure 3A and 3B). However, if the weak low-frequency stimulation occurs after another group of 100 synapses had been stimulated by the strong low-frequency protocol, then the group that has received the weak stimulation shows consolidated synapses (at 90±2 percent 5 hours after stimulus induction, Figure 3C). Moreover, consolidation of LTD (at 92±3 percent 5 hours after stimulus induction) in the group of synapses receiving the weak low-frequency protocol also occurs if it was stimulated thirty minutes after the stimulation of a second group of synapses by a strong tetanus, leading to LTP (Figure 3D). Thus, the TagTriC model exhibits cross-tagging consistent with experiments [11],[32]. In our model, cross-tagging occurs because the tags for LTP and LTD (*h_i_* and *l_i_*, respectively) enter in a symmetric fashion into the trigger criterion for the synthesis of plasticity-related proteins (see Figure 1 and Methods).

### Model Mechanism for Tagging, Cross-Tagging, and Consolidation

**Figure 4:**
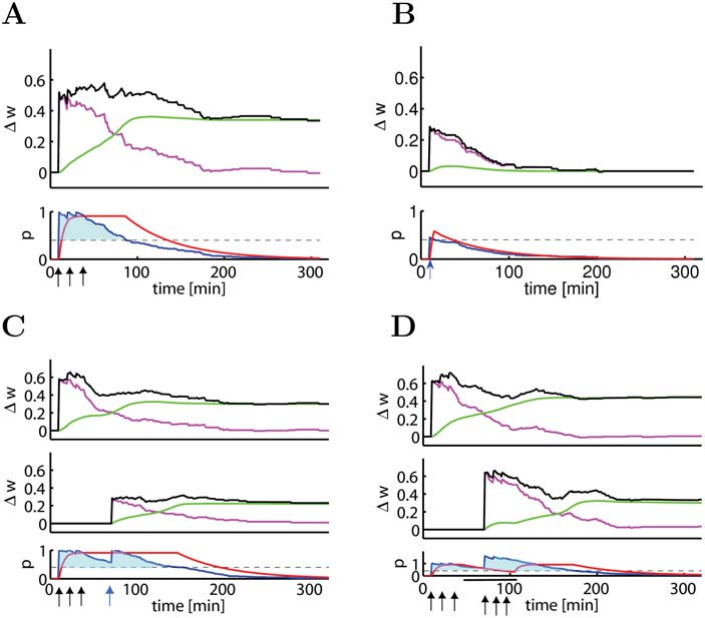
Dynamics of the TagTriC Model during different tagging protocols and protein synthesis blocking. The change of the total synaptic weight (top panels, black line 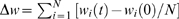) has contribution from early LTP (top panels, magenta line represents 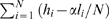) and from late LTP (top panels, green line represents 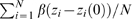). The protein variable *p* (red line, bottom panels) grows as long as the average number of tags (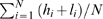, blue line) is above the protein synthesis trigger threshold (*N_p_*/*N*, dashed horizontal line). For better visibility, the regions where the blue line is above the trigger threshold is shaded. (A) A strong tetanus (*N* = 100 synapses, stimulated by 100 pulses at 100 Hz, repeated three times every ten minutes) leads to a sustained period of about 90 minutes where the number of tagged synapses is above the protein synthesis triggering threshold (lower panel, blue shaded). During this time the protein synthesis variable *p* is close to one (red line, lower panel), causing an increase in the fraction of consolidated weights (green line, top panel). (B) During a weak tetanus (*N* = 100 synapses, stimulated by 21 pulses at 100 Hz) the number of tags surpasses the protein triggering threshold only for a short time which does not enable switching of the *z* variable (top panel, green line) to the up-regulated state. (C) If the weak tetanus is given 30 minutes after the strong one, the number of tags set by the strong tetanus is still above the threshold, which allows protein synthesis stabilizing both the group of 100 synapses receiving the strong tetanus (top panel) and the group of 100 synapses receiving the weak tetanus (middle panel). (D) Protein synthesis is blocked for 1 hour (indicated by black bar at bottom of panel) starting 35 minutes after a first group of 100 synapses has been stimulated by a strong tetanus. Despite protein synthesis blocking, both the first group of synapses (top panel) and a second group of 100 synapses that received a strong tetanus during the blocking period (middle panel) develop late LTP because proteins synthesized during the induction of early LTP in the first group decay only slowly (bottom panel).

In order to elucidate how the model gives rise to the series of results discussed in the preceding paragraphs, we have analyzed the evolution of the model variables during and after induction of LTP (Figure 4). Critical for consolidation is the synthesis of plasticity related proteins, characterized by the variable *p* in the model. Synthesis is only possible while the total number of tags 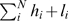 is above the protein triggering threshold *N_p_*. For the strong tetanic stimulus this criterion is met for about 90 minutes (shaded region in Figure 4A) leading to high levels of plasticity related proteins. After 90 minutes the concentration of proteins starts to decay back to baseline. While the level of proteins is sufficiently elevated the consolidation variable *z_i_* of each tagged synapse moves towards *z_i_*≈1 since this is the only stable fixed point of the dynamics (Figure 1C). This leads to a consolidation time of about 2 hours, enough to switch a large fraction of synapses into the up-regulated state *z*≈1 (green line, Figure 4A). Hence the average weight of the stimulated synapses stabilizes at a value above baseline, indicating L-LTP (Figure 4A, solid line).

**Figure 5:**
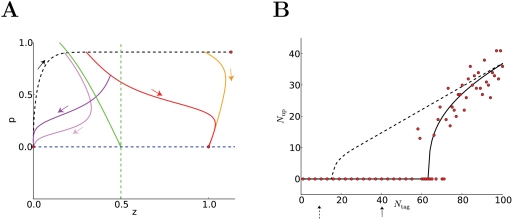
Theory and predictions. (A) Evolution of the variables *p* and *z* during tagging. If protein synthesis is ‘ON’ and the synapse tagged, *p* and *z* move along the black dashed line towards the stable fixed point on the upper right (*p*≈1, *z*≈1) (red filled circle). If protein synthesis stops after some time (yellow line, after 90 min; orange line, after 40 minutes) but the synapse remains tagged, the dynamics converges towards the fixed point *p* = 0, *z* = 1 (red filled circle) indicating that the synapse is consolidated (yellow and orange trajectories). However, if protein synthesis stops too early (after 25 min, pink line), or if the synaptic tag is lost too early (after 60 min, magenta line), the synapse is not consolidated and the trajectories converge towards the non-tagged initial state *p* = 0, *z* = 0 (red filled circle). The green dashed vertical line at *z* = 0.5 indicates the threshold beyond which a loss of the tag does not affect consolidation; the green solid line indicates the separatrix between the stable fixed points at *z* = 0 and *z* = 1. The minimal duration of protein synthesis to allow any consolidation is given by the intersection of the black dashed line with the separatrix. (B) Number of consolidated synapses (*N*_up_, vertical axis) as a function of the number of initially tagged synapses (*N*_tag_, horizontal axis) in simulations (red filled circles) and theory (solid line). Some of the initially tagged synapses fail to be consolidated because either they lose their tag or protein synthesis stops too early (see A). With a protein synthesis threshold *N_p_* = 40 (arrow) we need about 60 initially tagged synapses to achieve any consolidation (solid line). If the protein synthesis threshold is reduced to *N_p_* = 10 (dashed arrow), we need at least 15 tagged synapses to see any consolidation (dashed line).

If, in a different experiment, 100 synapses are stimulated by the weak tetanus, the synthesis of plasticity related proteins is only possible during a few minutes (Figure 4B, red line), which is not sufficient to switch tagged synapses from *z* = 0 into the upregulated state *z*≈1. Hence the weights (Figure 4B, black line) decay together with the tags (Figure 4B, magenta line) back to baseline and the transition from early to late LTP does not occur. The decay of the weights is controlled by the rate *k_H_* at which tags stochastically return to zero. The evolution of the protein concentration *p* and the consolidation variable *z* after a strong tetanus that leads to 90 minutes of protein synthesis and a weaker tetanus that only leads to 40 minutes of protein synthesis has been illustrated in (Figure 5A).

The total amount of available protein that is synthesized depends in our model on the time that the total number of tags stays above the protein triggering threshold *N_p_*. Even though always 100 synapses are stimulated in our model, not all receive tags in each experiment; moreover because of the competition for potentiation tags (*h_i_* = 1) and depression tags (*l_i_* = 1) during induction of plasticity, different synapses can receive different tags in the same experiment. With our strong tetanus protocol, on average 70 (out of 100) synapses receive a potentiation tag and 30 a depression tag while with the weak tetanus the numbers are 30 and 10, respectively. For the depression protocols, on average 10 synapses receive a potentiation tag and 90 a depression tag under strong low-frequency stimulation, and typically zero a potentiation tag and 40 a depression tag under the weak low-frequency protocol. These numbers vary from one trial to the next so that sometimes the protein trigger threshold *N_p_* = 40 is reached with the weak protocols and sometimes not. The important aspect is that even if the threshold is reached for a short time, the duration of protein synthesis is not long enough to provide a sufficient protein concentration *p* for consolidation of the tagged synapses; see Figure 4B and Figure 5A.

Since the concentration *p* of plasticity related proteins is crucial for the transition from early to late LTP we wondered how a block of protein synthesis would interfere with the consolidation of weights in the TagTriC model. Application of a protein synthesis inhibitor (modeled by setting the rate *k_p_* of protein synthesis to zero) during 1 hour starting thirty minutes before a strong tetanus is given to a group of 100 synapses that would normally lead to L-LTP, induced E-LTP but prevented consolidation into L-LTP (data not shown). However, if the same simulation experiment was repeated after a second group of synapses had received a strong tetanic stimulation 35 minutes prior to the application of protein synthesis blocker, then both groups of synapses showed consolidation of weights (Figure 4D), consistent with experiments [12]. Closer inspection of the lower panel in Figure 4D shows that two components contribute to consolidation: Firstly, the concentration of plasticity related proteins (red line) that has increased because of the first strong tetanic stimulus decreases only slowly back to baseline enabling the switching of the slow components (variable *z*, green line) even in the presence of protein synthesis blocker. Secondly, even after the end of the application of the blocker, the total number of tags that has been set by LTP induction is still above the critical value *N_p_* (shaded region in Figure 4D) so that protein synthesis can be resumed after the end of the blocking period. In summary, the detailed analysis of the TagTriC model allows to account for many aspects of tagging experiment in terms of a limited number of variables.

## Discussion

### Relation of Models to Experiments

Synaptic plasticity is based on intricate signal transduction chains involving numerous processing steps and a large number of different molecules [2],[13],[17]. Despite the complexity of the molecular processes, synaptic plasticity has experimentally been characterized by a small set of distinct phenomena such as short-term plasticity [44] as well as early and late phases of LTP and LTD [13].

Existing models of synaptic plasticity have focused on the description of short-term plasticity [44] and on the induction of LTP and LTD [24]–[26], [33]–[36]. The question of maintenance has received much less attention and was mainly addressed in the context of bistability of the CaMKII auto-phosphorylation process [27]–[29], AMPA receptor aggregation [41], or four identified kinase pathways [45]. While CaMKII is necessary for induction of long-term potentiation [46], it is probably too narrow to focus modeling studies only on a single or a few kinases such as CaMKII and neglect other proteins and signaling cascades that are involved in synaptic maintenance [13]. For example, there is strong evidence that PKM*ζ* is involved in synaptic maintenance and necessary for the late phase of LTP in vitro [11] and in vivo [14]. However, the actual processes are complex and the molecules involved in setting tags may differ between different parts of the dendrite. For example PKM*ζ* is involved in setting tags during E-LTP in the basal dendrite, whereas CaMKII (or MAPK for E-LTD) plays a similar role in apical dendrites [30].

Instead of focusing on specific signaling cascades, the TagTriC model presented in this papers aims at describing the essential ingredients of any possible functional model of L-LTP and tagging. These ingredients include (i) a bistable switch (described by the dynamics of the *z_i_*-variable) for each synapse that guarantees long-term stability in the presence of molecular turn-over [16]; (ii) a global triggering signal for protein synthesis (described by the dynamics of the *p* variable); a formalism to (iii) induce early forms of LTP and LTD and (iv) set synaptic tags. Since we aimed for the simplest possible model, we have identified the synaptic tags *h_i_* and *l_i_* for potentiation and depression with the synaptic weights during the early phase of LTP and LTD, respectively, so that points (iii) and (iv) are described by the same transition of the synapse from an initial non-tagged state to the high or low state, respectively. Variants of the model where the weight during the early phase of LTP and LTD is not directly proportional to the value of the tags are conceivable.

Even though we do not want to identify the synaptic variables *h_i_*, *l_i_*, *z_i_* with specific biochemical signals, a couple of candidate molecules and signaling chains should be mentioned. The setting of the tag for LTP under normal physiological conditions involves NMDA receptor activation and elevated levels of calcium which in turn trigger a signaling chain involving Calmodulin and CaMKII. We therefore think that the *h_i_* variable (representing both the tag for LTP induction and the weight increase during the early phase of LTP) should be related to the activation of CaMKII [13],[46]. The molecular interpretation of the tag *l_i_* for LTD is less clear [13]. In our model we have taken the tags as discrete quantities that decay stochastically, but a model with continuous tags that decrease exponentially gives qualitatively the same results (data not shown). The reason is that triggering protein synthesis in our model requires a large number of tags to be set, so that even in the stochastic model only the *mean* number of tags is relevant–and the mean (more precisely, its expectation value) is a continuous variable. Nevertheless, we prefer the model with discrete values over the continuous one in view of the switch-like transitions of synapses after induction of LTP and LTD [7],[37]. Maintenance of enhanced synaptic weights is probably implemented by an increased number of AMPA receptors in the postsynaptic membrane. Whether the stability arises from a self-organization process of receptors [41] or from interaction with persistently activated CaMKII molecules [46] or from additional kinases such as PKM*ζ* [11],[14], is an open problem of experimental investigation. Similarly, the exact identity of many plasticity related proteins is still unknown [13]. In our model we assume that recently synthesized plasticity related proteins are accessible to all synapses onto the same postsynaptic neuron. However, a distinction between proteins synthesized in, say, basal dendrites and that synthesized in apical dendrites would be possible by replacing the variable *p* by two or more distinct variables *p_k_* with similar dynamics (but potentially different trigger thresholds *N_p_*), allowing for a compartmentalization of tagging [13].

Experimental cross-tagging results clearly indicate that there are two different types of synaptic tags, one for LTP and one for LTD [13],[32], which we called *h_i_* for LTP and *l_i_* for LTD, leading to three different states during tagging (Figure 1A). Since we have identified the tagging with the early phase of LTP and LTD, our model of E-LTP and E-LTD also has three different states (whereas our model of late LTP/LTD has only two states characterized by *z_i_* = 0 and *z*_−_ = 1). The three-state model of early LTP/LTD presented in this paper would predict that all non-tagged synapses can undergo a transition to E-LTP or E-LTD depending on the induction protocol–whereas experiments suggest that about 70 percent of synapses show LTP but not LTD and the remaining 30 percent LTD but not LTP [7]. Moreover, only those synapses that are initially weak can be potentiated and only those that are initially strong can be depressed [7]. This aspect can be included in our model if we replace the induction rates *ρ_H_* for LTP by *ρ_H_*(1−*z_i_*) and *ρ_L_* for LTD by *ρ_l_z_i_* so LTP is only possible from a state with *z_i_* = 0 and LTD only from an initial state *z_i_* = 1 — in agreement with a two-state model of early LTP/LTD [7]. For the tagging and induction experiments presented in this paper, the results do not change significantly when we implement this extension of the induction model.

### Functional Consequences and Predictions

One of the advantages of a simple phenomenological model is that it should be capable of illustrating the functional consequences of tagging and L-LTP or L-LTD in a transparent manner. What are these functional consequences?

A characteristic feature that is made transparent in our model (and which we expect to be present in any model of tagging) is that, under typical experimental conditions, the transition from early to late LTP is only possible if a sizable group of synapses have undergone E-LTP or E-LTD. Hence, while induction of E-LTP is a local Hebbian process that is likely to take place at the postsynaptic site of the synapse (e.g., the dendritic spine), the transition from the early to the late phase of LTP requires a minimum number of synapses to be activated by appropriate stimulation including co-activation of neuromodulatory input so as to trigger synthesis of plasticity related proteins. A direct consequence of this is that synapses cannot be considered as independent. In order to predict whether a synapse memorizes an item for a long time or forgets it and re-learns some other item, it is not sufficient to consider a ‘Hebbian’ induction model, where synaptic changes depend only on the activity of pre- and postsynaptic neurons. For maintenance, it is not the synapse which decides individually, but it is the neuron as a whole (or a large functional compartment sharing the same site of synthesis of plasticity-related proteins [13],[30],[47]) which ‘decides’ whether it is going to store the present information, or not. Hence, classical [20],[21],[34] and recent [22] theoretical models which studied memory maintenance in the presence of ongoing neuronal activity on the level of *single* synapses need to be reconsidered, since the assumption of independent synapses does not hold (Figure 5A and 5B). In particular, our model predicts that, after an ensemble of identical neurons have received the same stimulus, some neurons learn (adapt a *large* fraction of their synapses to the stimulus) and others don't (keep all their synapses unchanged). With our choice of parameters, this happens in the TagTriC model if the number of synapses that have been tagged during the induction protocol is between 55 and 70 (Figure 5B). This neuronal, rather than synaptic, decision about memorizing an input (see also [48]) is potentially attractive for prototype learning–a standard paradigm in neuronal clustering and categorization algorithms, e.g., [19]. In contrast to traditional neuronal clustering models where learned memories need to be protected against overwriting by completely different memory items [19], a model based on tagging would have an intrinsic vigilance threshold via the trigger threshold *N_p_*. Hence it is resistant to changes at a single synapse.

In our view, the protein synthesis trigger threshold *N_P_* is an important control parameter in the model. The results of Figure 2F show that an increase of the trigger threshold reduces the maximal delay after which a weak tetanus leads to L-LTP after a strong tetanic stimulation in a different group of synapses. With our normal value of *N_p_* = 40 we need around 60 synapses to be initially tagged in order to retain any memory. If we decrease the trigger threshold to *N_p_* = 10 and keep all other parameters of the model unchanged, then we need at least a group of 15 synapses tagged during the induction protocol to get any consolidation since some of the initially tagged synapses loose their tag too early to get consolidated (Figure 5B). Only for a very small trigger threshold, say *N_p_* = 1, (which could occur at high concentration of neuromodulators) synapses become (nearly) independent, since a tag at a single synapse would be sufficient to trigger the synthesis of proteins which would then become available at that synapse. Repeated stimulation of the synapse alone would then be sufficient to transform E-LTP into L-LTP.

In our opinion, the trigger threshold *N_p_* is significantly lower in the presence of neuromodulators such as, for example, dopamine (for synapses from Schaffer collaterals onto CA1 pyramidal neurons) or noradrenaline (for synapses in the dentate gyrus). A simple model for the dependence of *N_p_* on dopamine would be *N_p_* = *n*_0_/(DA_bg_+*c*_0_) where *n*_0_ is some arbitrary number (say *n*_0_ = 1), *c*_0_ a small number (say 0.001) and DA denotes the stationary ‘background’ concentration of dopamine (that is, before the start of the experiment), normalized to 0<DA_bg_<1. The phasic dopamine signal caused by co-stimulation of dopaminergic input during tagging experiments is assumed to be proportional to the number of tags 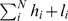. The trigger condition 
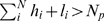
 becomes then equivalent to the condition 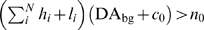 which shows a trade-off between the phasic dopamine signal and the stationary background level of dopamine. In particular in the presence of a large concentration of dopamine (DA≈1), single synapses can be consolidated. With the assumption that standard tagging experiments in a large group of synapses are performed at a low dopamine concentration of DA = 0.024 before stimulation, we retrieve the value of *N_p_* = 40 used in the main part of the results section. The dependence of the trigger criterion on the number of tags 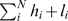 takes implicitly the co-activation of neuromodulatory input during the experimental stimulation protocol into account: the larger the number of stimulated neurons and the stronger the stimulus, the higher the probability of co-activation of dopaminergic fibers. Blocking dopamine receptors amounts in the model to setting both the background and the phasic dopamine signal to zero. In this case, protein synthesis is not possible.

Our model of LTP/LTD induction does not only account for voltage and frequency dependence of LTP/LTD induction, but also for spike timing dependence. In fact, for a stimulation paradigm where postsynaptic spikes are induced by short current pulses of large amplitude either a few milliseconds before or after presynaptic spike arrival, the model of LTP/LTD induction used in the TagTriC model becomes formally equivalent to a recent model of spike-timing dependent plasticity [35] which can be seen as an extension of classical models of STDP [24]–[26]. In the case of stochastic spiking of pre- and postsynaptic neurons our model shares important features with the Bienenstock-Cooper-Munro model [33], in particular the quadratic dependence upon the postsynaptic variables. In addition, our model also accounts for the voltage dependence of the Artola-Bröcher-Singer model [38]. Thus, the model of LTP/LTD induction shares features with numerous established theoretical models and covers a large range of experimental paradigms known to induce LTP or LTD [3]–[6],[8].

Since the subsequent steps of protein synthesis trigger and stabilization are independent of the way early phase of LTP is induced, our model predicts that tagging experiments repeated with different stimulation paradigms, but otherwise identical experimental preparation and age of animal, should give similar results as standard tagging protocols. In particular we propose to stimulate a group of synapses in hippocampal slices by 40–60 extracellular current pulses at 10 Hz while the postsynaptic neuron is receiving intracellular current injection that triggers action potential firing either a few milliseconds before or after presynaptic spike arrival and keeps the membrane potential at a depolarized level between postsynaptic action potential firing. Our model predicts that this will induce early LTD or LTP depending on spike timing and depolarization level that is not maintained beyond 1 or 2 hours. However, if the same stimulation occurs after a second group of synapses has received a strong tetanus, then stabilization of synapses at potentiated or depressed levels should occur, similar to standard tagging and cross-tagging experiments. In our opinion, these predictions should not depend on model details, but hold for a broad class of models that combine a mathematical description of induction of synaptic plasticity with a mechanism of consolidation.

Another finding—which is somewhat unexpected and in contrast to other conceptual models of synaptic tagging and capture [12],[13],[47]—is that during a strong tetanic stimulation a fraction of synapses receives tags for depression (while most, but not all, receive tags for potentiation). This is due to the fact that during induction of plasticity, transition to E-LTP and E-LTD act in parallel [7]. The prediction is that after consolidation (say 2 hours after the strong tetanic stimulation) a small fraction of synapses would show L-LTD, rather than L-LTP.

An essential ingredient of our model that allows long-term stability of consolidated synapses is the bistable dynamics of the variable *z*. In our opinion, such bistability (or possibly multistability [49] with three or four stable states) is necessary for synaptic maintenance in the presence of molecular turn-over, as recognized in earlier theoretical work [15],[16],[34]. Our model therefore predicts that L-LTP and L-LTD should have bistable, switch-like properties. While there is evidence for switch like transitions during the induction of E-LTP and E-LTD [7],[37], the bistability of the late phase of synaptic plasticity has so far not been shown. A possible experiment would be to combine a minimal stimulation protocol (e.g., a weak tetanus) at a single synapse [7],[37] with a medium to strong stimulus at a group of other synapses (e.g., tetanic stimulus varying between 30 and 100 pulses). The prediction is that the weight of the single synapse shows an all-or-none phenomenon with transition probabilities that depend on the stimulation of the group of other synapses. In particular, as the number of pulses of the tetanic stimulation is reduced (covering a continuum from strong to weak tetanic stimulation), the maintenance in the potentiated state should become less likely (averages across many experiments decrease) whereas the results of individual experiments show either full potentiation or none, which should give rise to a bimodal distribution of normalized synaptic weights.

### Open Questions and Perspectives

A lot of questions remain open and need to be addressed in future studies. First, can a synapse that has been potentiated in the past and is maintained after a transition to late LTP undergo a further potentiation step [13]? In our current model this is not possible since the consolidation variable *z* has only two stable fixed points. If we replace the function *f*(*z*) depicted in Figure 1 by another one with more than two stable fixed points, then the answer to the above question would be positive. Indeed, there have been suggestions that self-organization of receptors into stable sub-groups could lead to multiple stable states [49].

Second, induction of LTP or LTD is not only possible by strong extracellular stimulation of groups of synapses, but also at single synapses if presynaptic activity is paired with either a depolarization of the postsynaptic membrane [5],[7] or tightly timed postsynaptic spikes as in STDP experiments [6],[8]. How can it be that the change induced by STDP seems to be maintained over one hour without visible degradation? [6],[7]. Are synapses in these experiments consolidated, and if so what is the concentration of neuromodulators? In the TagTriC model with the choice of parameters used in the present paper, consolidation would not be possible, since the minimum number of synapses that have undergone E-LTP or LTD is *N_p_* = 40 in order to trigger protein synthesis, but, as explained above, an increased neuromodulator concentration would make consolidation possible.

Third, what is the role of NMDA receptor activation during synaptic consolidation? In our present model, protein synthesis is triggered by appropriate induction protocols, but is independent of synaptic activity during the consolidation process. However, recent experimental results suggest that protein synthesis blocker needs synaptic stimulation during the consolidation period to become effective [50], suggesting a subtle interplay between protein synthesis and synaptic activation that cannot be captured by our model.

Fourth, has each neuron a single protein synthesis unit or is protein synthesis a local process confined to each dendritic branch? In the first case, there is a single neuron-wide protein synthesis trigger threshold [12] and the neuron as a whole ‘decides’ whether early forms of synaptic potentiation and depression will be consolidated or not. This is the paradigm posited in the TagTriC model. In the alternative model of local protein synthesis [13],[47], the critical unit for consolidation are local groups of synapses on the same dendritic branch. Thus, for the same number of tagged synapses, a local group of synapses on the same dendritic branch is more likely to undergo consolidation than a distributed set of tagged synapses, leading to a form of clustered plasticity [47]. The TagTriC model can be easily adapted to the case of clustered plasticity by (i) replacing the point-neuron model by a neuron model with spatially distributed synapses and (ii) replacing the neuron-wide trigger equation (see 4 and Figure 1B) by a finite number of analogous, but dendrite-specific equations.

Fifth, how can tags be reset? Experiments show that a depotentiating stimulus given 5 minutes after a weak tetanus erases the trace of E-LTP (resets the tag) whereas depotentiation 10 or 15 minutes after the strong tetanus only transiently suppresses the E-LTP, making the consolidation of the synapse by protein capture possible [51]. We have checked in additional simulations that our present model cannot account for these experiments. In our opinion, the above tag-reset experiments show that the synapse has additional hidden states currently not included in the TagTriC model. Additional states would allow to (i) separate the measured early LTP during the first 5 minutes from setting the tag; and (ii) distinguish between depotentiation and depression of synapses. One interpretation of the tag-reset experiments [51] is that during the first five minutes the tag is not yet set whereas early LTP is already visible. The tag would be set only with a delay of 5–10 minutes. Application of a depotentiating stimulus more than 10 minutes later would then leave the potentiation tag intact, but move the synapse to a transiently depotentiated state.

The final and potentially most interesting question is that of functional relevance: Can the TagTriC model be used to simulate reward-based learning in experiments in vivo [13]? The formal theory of reinforcement learning makes use of an eligibility trace [52] which can be interpreted as a synapse specific tag. In the future we want to check whether the TagTriC model can be linked to reinforcement learning models [53]–[56] under the assumption that reward prediction errors are represented by a dopamine signal [57] which influences the protein synthesis dynamics in our model. This open link to reward-based learning is of fundamental functional importance.

## Methods

### Model of Early LTP/LTD and Tagging

In our model we assume that presynaptic spike arrival needs to be combined with a depolarization of the postsynaptic membrane (e.g., [5]) in order to induce a change of the synapse. In voltage clamp experiments (e.g., [39]) the postsynaptic voltage would be constant. However, in general the voltage is time-dependent and described by a variable *u*(*t*). In the TagTriC model, we assume that the low-pass-filtered voltage
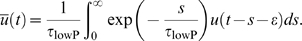
needs to be above a critical value *ϑ*_LTD_ to make a change of the synapse possible. *τ*_lowP_ is the time constant of the low-pass filter and *ε* = 1 ms is a short delay twice the width of a spike (see Table 1). This short delay ensures that *u̅* includes effects of previous presynaptic inputs and postsynaptic spikes, but not of an ongoing postsynaptic action potential.

**Table 1:** Parameter values used throughout all simulations, except Figure 1E–G where *N_p_* = 10 and initial percentage of *z_i_* = 1 was 10%, because these simulations refer to experiments with younger animals.

Tag	Trigger	Consolidation
*N* = 100	*k_p_* = 1/(6 min)	*N* = 100
*A*_LTD_ = 0.01	*τ_p_* = 60 min	*γ* = 0.1
*A*_LTP_ = 0.014	*N_p_* = 40	*τ_z_* = 6 min
*τ_x_* = 100 ms		*β* = 2
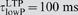		Initialisation: *N*(*z_i_* = 1) = 30
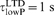		
ε = 1 ms		
*k_h_* = 1/h		
*k_l_* = 1/(1.5 h)		
Θ_LTD_ = −70.6 mV		
Θ_LTP_ = −50 mV		
*α* = 0.5		
Initialisation: *l_i_* = *h_i_* = 0		

Combining presynaptic spike arrival at synapse *i* (represented by *x_i_*) with a depolarization *u̅* of the postsynaptic neuron above a threshold *ϑ*_LTD_ we get a rate of LTD
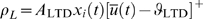
(1)where *A*_LTD_>0 is a parameter and [.]^+^ denotes rectification, i.e., [*y*]^+^ = *y* if *y*>0 and zero otherwise. Here 
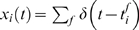
 denotes the presynaptic spike train with pulses at time 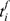 and *δ* the Dirac-delta function. Formally, *ρ_L_* describes the rate of stochastic transitions from the non-tagged state *h* = 0, *l* = 0 to the low state *l* = 1, Figure 1. In simulations we work with discrete time steps of Δ = 1 ms. Eq. 1 indicates that the probability *P_l_*_ = 0→*l* = 1_ of a transition to the low-state during the time step Δ vanishes in the absence of presynaptic spike arrival and takes a value of *P_l_*_ = 0→*l* = 1_ = 1−exp(−*A*_LTD_[*u̅*(*t*)−*ϑ*_LTD_]^+^Δ)≈*A*_LTD_[*u̅*(*t*)−*ϑ*_LTD_]^+^Δ if a presynaptic spike arrives at the synapse *i* during the time step Δ. Note that the transition from *l* = 0 to *l* = 1 is only possible if *h* = 0 and *h* remains zero during the transition.

Similarly, a switch from the non-tagged state *h* = 0, *l* = 0 to the high state *h* = 1 occurs at a rate *ρ_H_* which also depends on postsynaptic voltage and presynaptic spike arrival. We assume that each presynaptic spike at synapse *i* leaves a trace *x̅* *_i_* that decays exponentially with time constant *τ_x_*. The exact biophysical nature of the trace is irrelevant, but could, for example, represent the amount of glutamate bound to the postsynaptic receptor. The value of the trace at time *t* caused by earlier spike arrivals at time 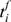 is then 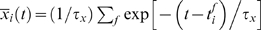 where the sum runs over all firing times 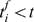. With the trace *x̅* *_i_* we write
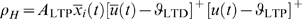
(2)which indicates that, in addition to the conditions for LTD induction we also require the *momentary* membrane potential *u*(*t*) to be above a second threshold *ϑ*_LTP_. This threshold could change on the time scale of minutes or hours as a function of homeostatic processes. To summarize, the rate of LTP transition *ρ_H_* is different from *ρ_L_* in five aspects. First, the constant *A*_LTP_ is not the same as *A*_LTD_. Second, LTP is caused by the *trace x̅* *_i_* left by presynaptic spikes, rather than the spikes themselves. This trace-formulation ensures that presynaptic spikes can interact with later postsynaptic spikes as in classical models of STDP [24]–[26]. Third, the time constant of the low-pass filter in *u̅* is different; fourth, the momentary voltage needs to be above a threshold *ϑ*_LTP_; and fifth, the total dependence upon the postsynaptic voltage is quadratic, rather than linear. The quadratic dependence ensures that for large depolarization LTP dominates over LTD [39]. Tagged synapses with *h_i_* = 1 decay with probability *P_h_*_ = 1→*h* = 0_ = *k_H_*Δ back to the non-tagged state (and analogously, but with rate *k_L_* for the transition *l_i_* = 1→*l_i_* = 0).

In the TagTriC model, the local synaptic values *h* = 1 for potentiation or *l* = 1 for depression act as tags indicating potential sites for further consolidation, but are also directly proportional to the weight of the synapse after induction of LTP or LTD. Since in minimal stimulation experiments LTD leads to a reduction of about 50 percent of the synaptic efficacy whereas LTP leads to an increase by up to 100 percent [7], we model the weight change during the early phase of LTP as Δ*w_i_* = (*h_i_*−*αl_i_*)*w̅* where *w̅* is the weight of the non-tagged synapse and *α* = 0.5. The total weight change Δ*w*/*w̅* measured shortly after induction of LTP or LTD with extracellular protocols corresponds to the fraction of synapses in the high or low states, respectively, hence, if all synapses start from the non-tagged state the measured weight change is 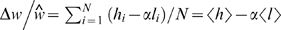 where *N* is the number of synapses stimulated by the protocol. The set of parameters of LTP/LTD induction and tagging is given in table 1.

### Trigger

The triggering process is controlled by the dynamics of a variable *p* which describes the amount of plasticity related proteins synthesized in the postsynaptic neuron. Protein synthesis is triggered and the variable *p* increases while the concentration of dopamine exceeds a critical level *ϑ_p_* [58]. If the dopamine concentration DA falls below *ϑ_p_*, the protein concentration decays with a time constant *τ_p_*. Assuming standard first-order kinetics we have
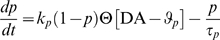
(3)Protein synthesis has a maximum rate *dp*/*dt* of *k_p_* and saturates if the amount of protein approaches a value one. Θ[*y*] denotes the unit step function with Θ[*y*] = 1 for *y*>0 and zero otherwise.

Dopamine is present at a low stationary background value. In addition a phasic dopamine component is induced in standard tagging experiments in hippocampal slices, because of co-stimulation of dopaminergic inputs during extracellular stimulation of presynaptic fibers [40]. To describe the time course of the phasic dopamine component in our model, we assume that the dopamine is proportional to the total number of tags Σ*_i_*(*h_i_*+*l_i_*) induced by the stimulation protocol. The stationary background level of dopamine DA_bg_ is included in the threshold *ϑ_p_* = *N_p_*(DA_bg_) for protein synthesis. Hence Eq. 3 can be rewritten in the form
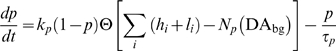
(4)Note that we have chosen units so that the threshold for protein synthesis *N_p_* can be interpreted as the minimal number of tags necessary to stimulate protein synthesis. This interpretation is important for the discussion of the model results, in particular Figures 4 and 5.

A suitable model for dependence of the protein synthesis threshold on the background level of dopamine is *N_p_*(DA_bg_) = *n*_0_/(DA_bg_+*c*_0_) where *n*_0_ = 1 is a scaling factor, *c*_0_ = 0.001 a constant and 0≤DA_bg_≤1 is the normalized dopamine concentration. We note that the trigger condition [Σ*_i_*(*h_i_*+*l_i_*)−*N_p_*(DA_bg_)]>0 is then equivalent to the condition (DA_bg_+0.001)[Σ*_i_*(*h_i_*+*l_i_*)]>1. This formulation shows that there is a trade-off between background levels and phasic dopamine. Unless stated otherwise we always use in the simulation a fixed dopamine level DA_bg_ = 0.024 so that *N_p_* = 40. The specific model *N_p_*(DA_bg_) of the dependence upon background dopamine levels is therefore irrelevant.

We assume that the plasticity related protein *p* synthesized in the postsynaptic neuron is diffused in the dendrite of the postsynaptic neuron and hence available to all the synapses under consideration. Hence, the tags *h_i_* and *l_i_* have indices, since they are synapse-specific, whereas *p* in Eq. 4 does not.

### Consolidation and Late LTP

The consolidation variable *z* describes the late phase of LTP and follows the dynamics
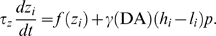
(5)


The scaling factor *γ* is a function of the dopamine level *DA*. In the simulations we always assumed a fixed dopamine level and set *γ*(DA) = 0.1.

In the absence of plasticity related proteins (*p* = 0), or if no tags are set (*h_i_* = *l_i_* = 0), the function *f*(*z*) = *z*(1−*z*)(*z*−0.5) generates a bistable dynamics with stable fixed points at *z* = 0 and *z* = 1 and an unstable fixed point at *z* = 0.5 marked by the zero crossings of the function *f*, Figure 1C. In the presence of a finite amount of proteins *p*>0 and a non-zero tag, the location of the fixed points changes and for *p*>0.47, only one of the stable fixed points remains. The potential shown in Figure 1C is a function *E* with *dE*/*dz* = −*f*(*z*) so that *dz*/*dt* = −*dE*/*dz*. We note that a synapse *i* can change its consolidated value only if both a tag (*h_i_* = 1 or *l_i_* = 1) and protein *p*>0.47 is present–summarizing the essence of ‘synaptic tagging and capture’ [12],[13].

### Synaptic Weight

The synaptic weights have contributions from early and late LTP and LTD. The total synaptic weight of a synapse *i* is *w_i_* = *w̅*(1+*h_i_*−*αl_i_*+*βz_i_*) where *w̅* is the value of a non-tagged synapse, *α* = 0.5 and *β* = 2 are parameters, *h_i_* and *l_i_* are binary values indicating E-LTP and E-LTD, respectively, and *z_i_* is the value of the L-LTP trace of synapse *i*. Since we model slice experiments in animals older than 20 days, we assume that 30 percent of the synapses have undergone previous potentiation and have *z* = 1 while the remaining 70 percent of synapses are in the state *z* = 0 [7]. In all simulation experiments we stimulate one or several groups of *N* = 100 synapses each. Assuming that no tags have been set in the recent past (*h_i_* = *l_i_* = 0), the initial value of the average weight in a group of *N* synapses is then 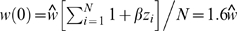.

### Neuron Model

For all simulations in this paper we use the adaptive exponential integrate-and-fire model [42] as a compact description of neuronal firing dynamics. Briefly, it consists of two equations. The voltage equation has an exponential and a linear term as measured in experiments [59]. The second equation describes adaptation. Although firing rate adaptation is not important for the present study, it would be relevant in the context of other stimulation paradigms. Parameters for the neuron model are as in [42] and are kept fixed for all simulations presented in this paper. The voltage threshold *V_s_* of spike initiation by a short current pulse is 25 mV above the resting potential of −70.6 mV [42]. Synaptic input is simulated as a short current pulse. The initial connection weight *w̅* was adjusted so that simultaneous activation of 40 or more synapses triggers spike firing in the postsynaptic neuron. Hence the amplitude of a single EPSP is about 0.6 mV.

The adaptive exponential integrate-and-fire model is defined in continuous time. If a spike is triggered by a strong current pulse, the voltage rises within less than 0.5 millisecond to a value of 20 mV where integration is stopped. The voltage is then reset to resting level, and integration restarted after a refractory time of 1 ms. In order to enable us to perform simulations of plasticity experiments with a time step of Δ = 1 ms, the voltage equation during the rising slope of the action potential was integrated once at a much higher resolution (time step 0.02 ms), so as to determine the exact contribution of each postsynaptic spike to the probability of LTP induction. Every postsynaptic spike was then treated as an event in the plasticity simulations that contributed a probability *P_h_*_ = 0→*h* = 1_ of flipping the tag from *h* = 0 to *h* = 1 in a time step Δ = 1 ms which we can write as *P_h_*_ = 0→*h* = 1_ = *a*_Δ_ *x̅*(*t*)[*u̅*(*t*)−*ϑ*_LTD_]^+^ with a numerical conversion factor *a*_Δ_ = *A*_LTP_ 5 ms mV derived by the above procedure; see Eq. 2.

### Number of Consolidated Synapses

In Figure 5 we plot the number of synapses that have been consolidated as a function of the number *N*_tag_ of initially tagged (*h_i_* = 1) synapses. Since the number of tags decays exponentially with rate *k_H_*, the expected duration 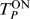 of protein synthesis is 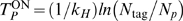 where *N_p_* is the protein trigger threshold. While protein synthesis is ‘ON’ the variables *p* and *z* move along the black dashed line in Figure 5A which crosses after a time *t*_1_ the separatrix (green line in Figure 5A) and at a time *t*_2_ the line *z* = 0.5 (vertical dashed green line). Different cases have to be distinguished. (i) 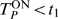, no consolidation takes place (see pink trajectory), hence *N*_up_ = 0. (ii) 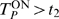, consolidation is guaranteed for all synapses that are still tagged at time *t*_2_, hence *N*_up_ = *N*_tag_exp(−*kt*_2_). (iii) In the case of 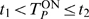, the time *t*_cross_ needed to cross the vertical line *z* = 0.5 is numerically calculated by integrating the equations *dp*/*dt* = −*p*/(*τ_p_*) and *dz*/*dt* = *f*(*z*)+*γ p* starting at 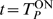 at the point 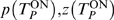 on the black-dashed line (see orange line in Figure 5A for a sample trajectory). The number of consolidated synapses is then *N*_up_ = *N*_tag_exp(−*kt*_cross_). The solid line in Figure 5B represents *N*_up_ as a function of *N*_tag_ calculated for the cases (i)–(iii). With our standard set of parameters, we have *t*_1_≈28 min and *t*_2_≈60 min.
